# Editorial

**DOI:** 10.1093/gbe/evac027

**Published:** 2022-02-27

**Authors:** Adam Eyre-Walker, Laura A Katz

**Affiliations:** Editors-in-Chief, Genome Biology and Evolution

We are now entering our fourth year as Editors-in-Chief (EiC) of *Genome Biology and Evolution* and we thought we would take this chance to look back at how the journal has evolved over these last few years. When we took over from Bill Martin, who had been EiC for the previous 10 years, the journal was in very good shape. We still continue to receive around 500 manuscripts a year, of which we publish about 250. These papers cover a broad range of topics, from the data-rich papers on genome evolution in microbial lineages, to studies of human population genomics and the evolution of photosynthesis among eukaryotes, to newly developed methodologies for analyzing genome-scale data. We also have a growing number of reviews and perspectives that synthesize results from across fields and systems. We consider ourselves to be an inclusive journal, publishing papers on genome evolution from any species so long as the science is sound, and the question is interesting and has an evolutionary focus. 

We have also launched several initiatives. As a society journal, we felt it was important to publish science irrespective of the authors’ ability to pay, and we have developed a waiver policy that reflects this—if authors do not have the funds to pay the article processing charges, then we ask authors to submit a waiver request regardless of their geographic location or seniority. Our sister journal, *Molecular Biology and Evolution*, shares the same policy, and all waiver decisions for both journals are now handled by a committee that is independent of the editorial boards. Both journals are owned by the Society of Molecular Biology and Evolution (SMBE), and the income generated from the journals goes to support these waiver requests as well as the activities of the society, including subsidizing the annual meeting as well as satellite and regional meetings.

**Fig. 1.**
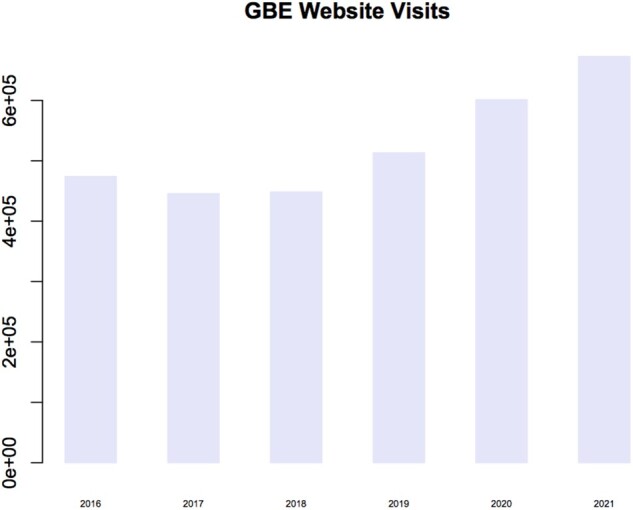
—Number of visits to the *GBE* website since 2016. Casey McGrath, the social media editor, was appointed in March 2019.

Early on in our tenure, we appointed a social media editor, Casey McGrath. As a science writer with a background in evolutionary biology, she writes highlight articles about our content and manages our social media accounts. We have seen a 50% increase in traffic to our website as a consequence of her efforts, and articles covered by her highlights see a significant boost in interest (fig. 1).

This year saw the publication of our first Special Section on “Within-individual genome variation and germline/soma distinction.” Special Sections are collections of papers on a particular topic. This has been followed by a Special Section and Virtual Issue on the “Phenotypic effects of synonymous mutations,” and we have just published one on “Comparative population genetics.” Several of these arose out of symposia from the annual SMBE conference. We now offer the chance for all symposium organizers to propose a Special Section, Review or Perspective from their symposium; we will extend this invitation to organizers of satellite and regional meetings when these recommence this year. We also welcome suggestions for Special Sections, Reviews, and Perspectives from the readership; if you have a good idea then please approach us.

Along with the Special Sections, we have been publishing Virtual Issues, in which we collate papers published in *GBE* over the previous 2 or 3 years on a particular topic. We have published Virtual Issues on *Human Genetics*, *Computational Biology*, and *Viral Evolution*. The papers published in these issues see a substantial increase in website traffic as a consequence of the edition. We have also initiated a biography section of the journal with the aim to highlight the many paths that scientists take in their career with a particular focus on scientists from disadvantaged backgrounds. We were honored to feature Wen-Hsiung Li, who has contributed so much to our understanding of molecular evolution, in our first biography. It has, however, turned out to be quite hard to get other scientists to write their biography—they are all too busy. So, we will be changing the style of these articles to an interview format. We hope that these pieces will serve as inspiration for the next generation of scientists and encourage students from all backgrounds to engage in a scientific career.

*GBE* is committed to inclusion and diversity. Currently, 31 (56%) of our 55 associate editors are women, and we have been working hard to increase ethnic and geographic diversity on the editorial board. We have also conducted our first audit of acceptance and rejection rates with regard to gender. We analyzed all manuscripts submitted to *GBE* in the calendar years 2016–2020 (inclusive). We used the genderize.io software (https://genderize.io/; Wais 2016), which uses social media information, to predict gender. We were able to predict gender, at a 70% confidence level, for 2092 first authors and 2335 last authors; the remaining authors had names that were difficult to assign gender to, either because they are ambiguous (e.g. Sam) or because there are insufficient data.

**Fig. 2.**
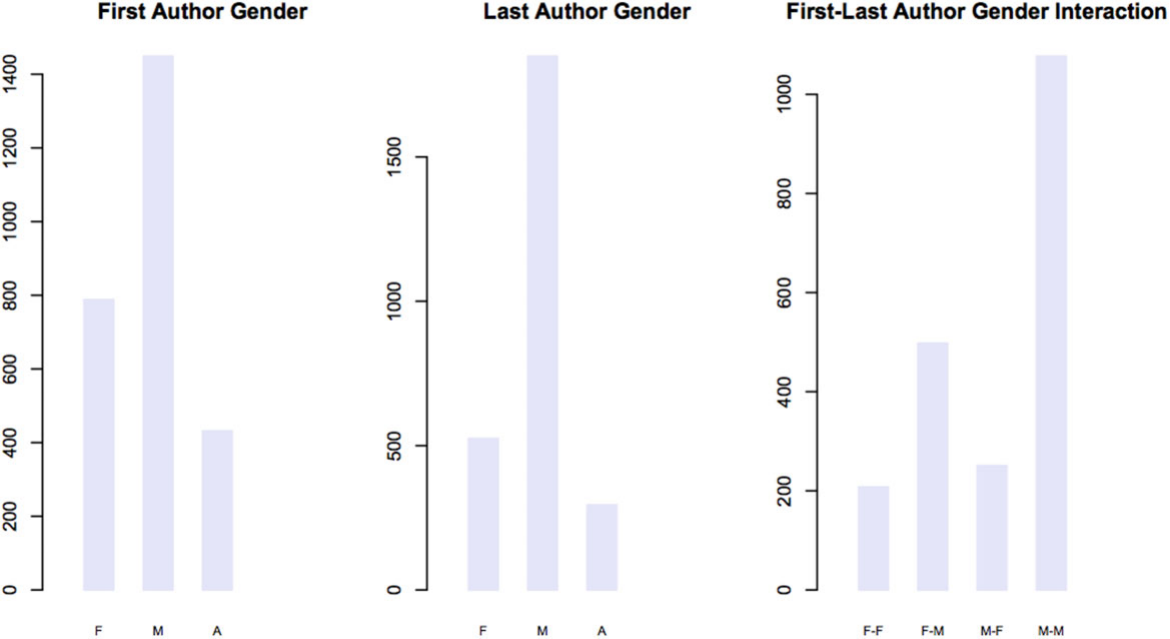
—The number of manuscripts submitted to *GBE* since 2016 that were authored by female, male, and authors of ambiguous gender, as either first or last author. In the interaction plot, the order is first–last author (e.g. F–M is a manuscript authored by a female first and male last author).

Overall, *GBE* receives significantly fewer manuscripts with female first and last authors (χ^2^ test, *P* < 0.001 in each case); we found that 29% of first authors and only 19% of last authors were female (fig. 2). The higher proportion of females amongst first relative to last authors might reflect an increase in the number of females pursuing scientific careers and/or the difficulty faced by women in obtaining a permanent position. We also find an association between the inferred genders of first and last authors on the same paper, with first authors found more frequently coauthoring papers with last authors of the same inferred gender (fig. 2). We intend to work to invite submissions of Research Articles, Reviews, and Perspectives, with an eye toward addressing this disparity in submission rates.

**Fig. 3.**
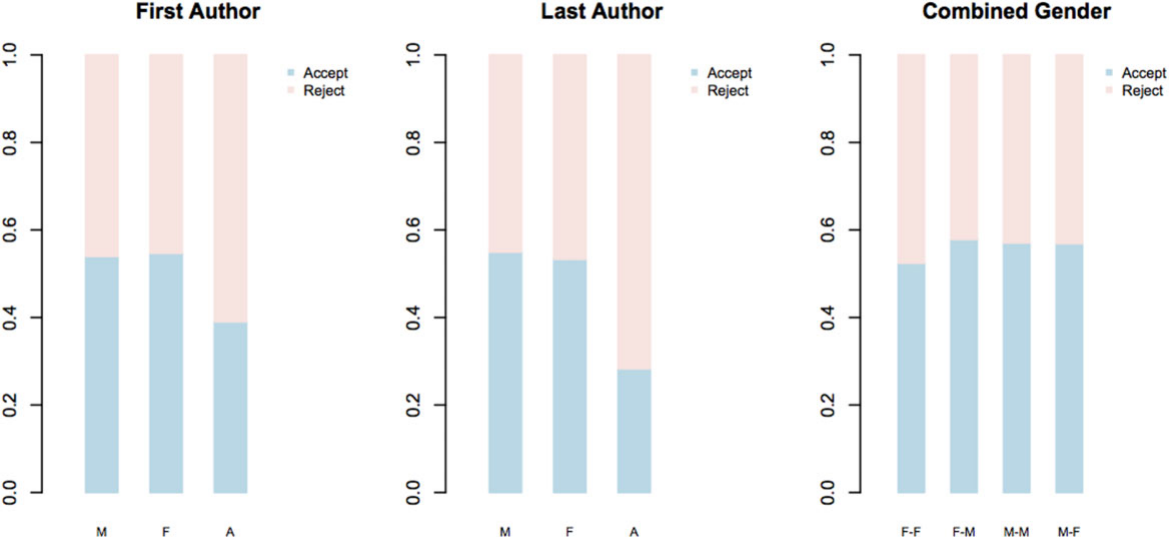
—The proportion of manuscripts that were accepted or rejected as a function of gender of the first, last, and combination of first and last authors. In the interaction plot, the order is first–last author (e.g. F–M is a manuscript authored by a female first and male last author).

In contrast to the bias in submission rates, we find that acceptance rates are the same for female and male authors, independent of whether they are first or last authors (χ^2^ tests: *P* = 0.80 for first authors; *P* = 0.56 for last authors, fig. 3)—acceptance rates amongst all authors are ∼54%. There is also no significant difference in the acceptance rates amongst the various gender combinations of first and last authors (χ^2^ test: *P* = 0.41; fig. 3). However, manuscripts with authors with names that we could not reliably assign gender to are significantly less likely to have their manuscripts accepted, whether they are first or last authors (χ^2^ tests versus combined male and female data *P* < 0.001 in all cases, fig. 3). As we move forward, we will continue to look at trends in these rates with a commitment to working toward removing bias.

Finally, we would like to thank the readership for reading the papers we publish, and the authors for submitting their work to us. We are proud of the journal and the society it serves. 
